# Epidermal Growth Factor Pathway in the Age-Related Decline of Oligodendrocyte Regeneration

**DOI:** 10.3389/fncel.2022.838007

**Published:** 2022-03-17

**Authors:** Andrea D. Rivera, Kasum Azim, Veronica Macchi, Andrea Porzionato, Arthur M. Butt, Raffaele De Caro

**Affiliations:** ^1^Department of Neuroscience, Institute of Human Anatomy, University of Padua, Padua, Italy; ^2^Department of Neurology, Medical Faculty, Heinrich-Heine-University, Düsseldorf, Germany; ^3^School of Pharmacy and Biomedical Science, University of Portsmouth, Portsmouth, United Kingdom

**Keywords:** EGF, EGFR, ErbB, oligodendrocyte, myelin, aging, white matter

## Abstract

Oligodendrocytes (OLs) are specialized glial cells that myelinate CNS axons. OLs are generated throughout life from oligodendrocyte progenitor cells (OPCs) *via* a series of tightly controlled differentiation steps. Life-long myelination is essential for learning and to replace myelin lost in age-related pathologies such as Alzheimer’s disease (AD) as well as white matter pathologies such as multiple sclerosis (MS). Notably, there is considerable myelin loss in the aging brain, which is accelerated in AD and underpins the failure of remyelination in secondary progressive MS. An important factor in age-related myelin loss is a marked decrease in the regenerative capacity of OPCs. In this review, we will contextualize recent advances in the key role of Epidermal Growth Factor (EGF) signaling in regulating multiple biological pathways in oligodendroglia that are dysregulated in aging.

## Introduction

**Figure 1:**
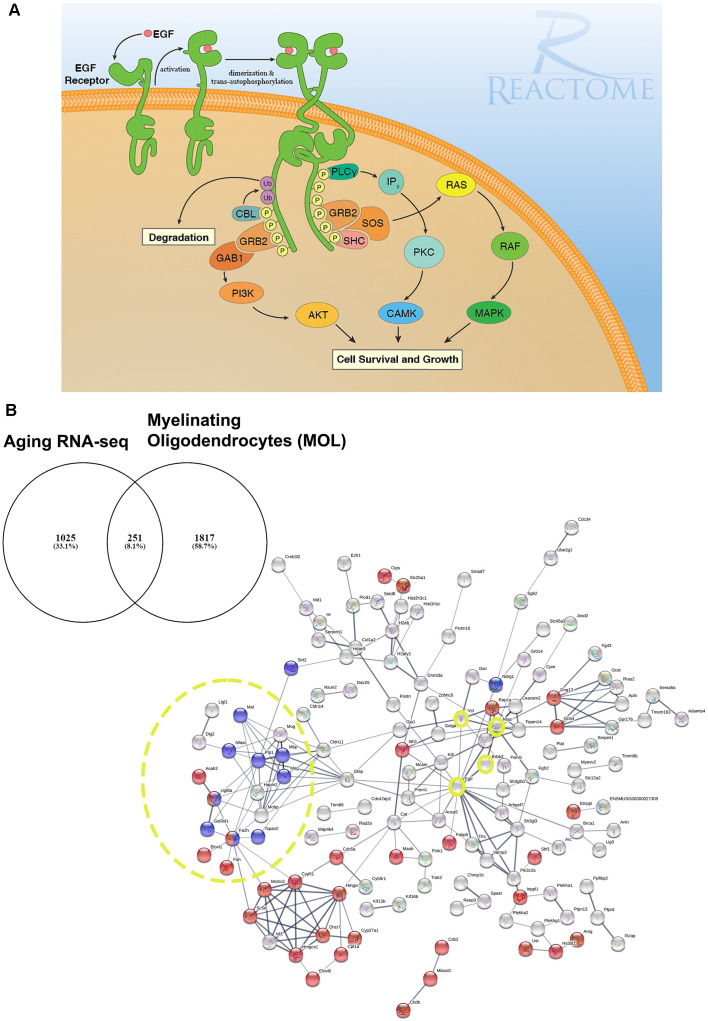
EGF receptor signaling and resolving its dysregulation in aged oligodendroglial *via* protein-protein network analysis. **(A)** EGFR is a member of ERBB receptors that belong to the superfamily of Receptor Tyrosine Kinases (RTKs). The binding of ligands to EGFR induces conformational changes resulting in the receptor homo- or heterodimerization at the cell surface. Dimerization of the extracellular regions of EGFR cascades results in further conformational change at the cytoplasmic region of the receptor, leading to the activation of the catalytic domain. EGFR dimers trans-autophosphorylate on tyrosine residues in the cytoplasmic tail becoming binding sites for the recruitment of intracellular modulator for downstream signaling cascades. Recruitment of complexes containing GRB2 and SOS1 directly through GRB2 or indirectly through SHC1 promotes the activation of RAS/RAF/MAP kinase signaling. The binding of GRB2 and GAB1 to phosphorylated EGFR leads to the activation of PI3K/AKT signaling cascade. Finally, PLCγcan be recruited to the phosphorylated EGFR which, in turn, activates IP3/PKC signaling. Image generated from REACTOME “Signaling by EGFR” (https://reactome.org/PathwayBrowser/#/R-HSA-177929). **(B)** RNA-seq transcriptome analysis of the aging murine brain was compared to a database of genes expressed by myelinating OLs (MOL) and 251 genes were identified as significantly altered in aging (Rivera et al., 2021b). **(B)** Functional protein-protein network analysis identified EGFR as centrally connected with ERBB3, NRAS, VCL, GSN, CLDN11, and the myelination node (yellow circles). Red nodes represent genes associated with Metabolism (*p* < 0.000034) and blue nodes represent genes associated with Myelination (*p* < 5.55e-07). PPI enrichment p-value < : 2.44e-15. Adapted from Rivera et al. (2021b).

Brain aging is characterized by a slowing down of sensory, cognitive and behavioral processes (Harada et al., 2013). Notably, brain imaging studies in humans have demonstrated shrinkage of white matter that precedes overt loss of neurons and appears to be accelerated in dementia (Bartzokis et al., 2012; Maniega et al., 2015; Cox et al., 2016). White matter is comprised of myelinated axons which are thin protrusions of the neurons that transmit electrical signals between the different parts of the central nervous system (CNS). Myelin is a lipid-rich insulating layer that is wrapped around axons in concentrical lamellae by terminally differentiated glial cells called oligodendrocytes (OLs). Myelin increases the propagation speed of electrical signaling along the length of an axon by saltatory conduction. Moreover, myelin has numerous emerging roles that includes, providing metabolic support (Fünfschilling et al., 2007; Philips and Rothstein, 2017), memory consolidation (Pan et al., 2020; Steadman et al., 2020), task-associated learning experiences (Kato et al., 2020; Wang et al., 2020), and reviewed by (Pan and Chan, 2021), whilst myelin loss renders axons more vulnerable to damage (Smith, 2006). Once developmental myelination is complete, myelin remodeling continues throughout life *via* a reservoir of oligodendrocyte progenitors (OPCs) which are the main proliferating pool of cells in the adult CNS and possess the stem-cell-like feature of self-renewal (Nishiyama et al., 2021). The life-long generation of OLs from OPCs is essential to produce new myelin required to insulate new brain connections formed in response to new life experiences and to replace myelin lost through natural “wear-tear” or pathology (Rivera et al., 2016). However, the regenerative power of OPCs declines with age leading to impaired oligodendrogenesis and myelin remodeling, and an overall gradual loss of major CNS functions such as spatial learning and memory (Pan et al., 2020; Steadman et al., 2020; Wang et al., 2020). The age-related impairments in OPC differentiation have been discussed in a number of recent reviews (for example, Rivera et al., 2021a; Butt et al., 2019). Moreover, in many age-related neuropathologies such as AD or secondary progressive MS, due to a number of reasons that include and are not limited to the inflammatory environment, excess inhibitory myelin debris, lack of appropriate trophic support, etc, OPC differentiation drastically fails and contributes to the loss in cognitive function (Neumann et al., 2019; Wang et al., 2020; Coelho et al., 2021; Rivera et al., 2021a,b). Currently, the development of treatments to halt these changes is hampered by gaps in fundamental scientific knowledge. Developmental studies propose a positive role of epidermal growth factor (EGF) acting *via* its main receptor, EGFR, as a key regulator of cell survival, proliferation, migration and differentiation which are disrupted in aging (Figure 1; Herbst, 2004; Gonzalez-Perez et al., 2009; Galvez-Contreras et al., 2013; Yang et al., 2017). To the best of our knowledge, functional studies of EGF signaling in the context of OL differentiation during later stages of adulthood have yet to be performed. Nevertheless, these are exciting future avenues in the field as a potential therapeutic target in OL pathologies and aging.

## Oligodendrocyte and Myelin Disruption in The Aging Brain

Age-related loss of brain connectivity underlies cognitive decline, with a “last in, first out” pattern, whereby white matter tracts associated with cognition are the “last” to be fully myelinated and the first to be lost in aging (Davis et al., 2009; Bartzokis et al., 2012; Gozdas et al., 2020). This process is the result of brain architectural complexity described as heterochronicity and spatial heterogeneity intrinsic in white matter tracts. In addition, it suggests that the latest tracts to develop are the most vulnerable to the deleterious effects of aging (Cox et al., 2016; Kochunov et al., 2016). Post-mortem diffusion magnetic resonance imaging (dMRI) studies indicate ontogenetic differences between early-myelinating projection and posterior callosal fibers in aging (Sexton et al., 2014; Cox et al., 2016; Slater et al., 2019). Although the precise causes of WM shrinkage are currently unresolved, they include metabolic disruption of oligodendroglia, OPC senescence and loss of extracellular trophic factors that support OPCs and OLs which can contribute to the functional decline of brain function including deficits in spatial memory and learning (Rivera et al., 2016; 2021b; Neumann et al., 2019; Kato et al., 2020; Pan et al., 2020; Steadman et al., 2020; Willis et al., 2020). Several studies in both humans and rodents have demonstrated marked changes in the transcriptome of OLs and myelination processes (Soreq et al., 2017; Neumann et al., 2019; Rivera et al., 2021b). Moreover, alterations in OPC densities have been reported in brain aging (Soreq et al., 2017; Rivera et al., 2021b). The age-related disruption of indispensable signaling pathway components hinders myelin remodeling and repair, and ultimately adds to the cumulative loss of myelin, which is aggravated in pathology. Recently, we have demonstrated that the critical OPC protein GPR17 is downregulated in the aged murine brain, together with myelin-related transcripts such as MBP, PLP1, CNP, and UGT8A (Rivera et al., 2021b). Our transcriptomic analysis identified a central role for age-related changes in EGFR signaling in oligodendroglia, consistent with its recognized importance in OLregeneration and myelin repair (Aguirre et al., 2007; Hayakawa-Yano et al., 2007; Ivkovic et al., 2008).

### Unraveling Novel Roles of EGFR Signaling in Aged Oligodendroglia

In our network analyses (Figure 1B), we identified EGFR association with myelination *via* its interaction with ERBB3 which is required for OL maturation (Schmucker et al., 2003; Makinodan et al., 2012). ERBB3 is coupled to the Ras family member NRAS which has intrinsic GTPase activity and is involved in the control of cell proliferation, regulating microtubule stability and actin polymerization (Fotiadou et al., 2007), and is implicated in cancer pathways (Bronte et al., 2015). Interestingly, NRAS has recently been reported to be elevated in expression in the aging OPC proteome (de la Fuente et al., 2020). NRAS interacts with VCL (vinculin) and CLDN11 (claudin-11) to regulate OL morphogenesis/myelin growth (Nawaz et al., 2015), or the formation of tight junctions (TJs) with ECM-integrin interactions, respectively (Gow et al., 1999; Bronstein et al., 2000; Tiwari-Woodruff et al., 2001). Notably, EGFRs are mechanosensitive (Tschumperlin, 2004; Müller-Deubert et al., 2017), transduced by vinculin (including Talin and similar linker proteins) to regulate the anchoring of the actin cytoskeleton to the ECM through integrin, leading to cytoskeleton regulation and cellular spreading (Rübsam et al., 2017). Our analysis predicted the interaction of vinculin on with the actin cytoskeleton *via* gelsolin (GSN), which is enriched in OLs (Tanaka and Sobue, 1994; Zhang et al., 2014) and is required for myelination (Liu et al., 2003; Zuchero et al., 2015). Intriguingly, vinculin and gelsolin are focal adhesion proteins important for the association of cell-cell and cell-matrix junctions and are critical for controlling cell spreading, cytoskeletal mechanics, and lamellipodia formation (Ciobanasu et al., 2014; Elosegui-Artola et al., 2016; Argentati et al., 2019; Merkel et al., 2019; Muñoz-Lasso et al., 2020). Moreover, the interaction of EGFR and vinculin with CLDN11 is consistent with the evidence that they mediate cell/integrin/ECM interactions (Hagen, 2017). Recent *in vivo* experiments in which CLDN11 was deleted in OLs have shown dysregulation of myelin with subsequent aberrant behavioral changes due to increased latency of signals (Maheras et al., 2018). The ECM plays a pivotal role in OL differentiation (Lourenço and Grãos, 2016) and increased stiffness of the ECM is related to age-related deterioration of OPC function (Segel et al., 2019). Our data implicate for the first time the EGFR-VINCULIN-GELSOLIN-CLDN11 network as key to age-related changes in oligodendroglial ECM interactions.

## EGFR and Their Roles in Oligodendroglia

### Overview of EGFR Ligands and Receptors in the CNS

The EGFR, also known as ERBB1 or HER-1, and its family of ligands are widely expressed across the CNS. EGFR, together with ERBB2, ERBB3, ERBB4 belong to the receptor tyrosine kinases (RTKs) superfamily (reviewed extensively elsewhere, for example, Novak et al., 2001; Fu et al., 2003; Galvez-Contreras et al., 2013). Canonical ligands include: epidermal growth factor (EGF), transforming growth factor-α (TGFα), Heparin-binding EGF (HB-EGF) B-cellulin (BTC) and low-affinity binding ligands such as neuregulins (NRG 1–4) amphiregulin (AR) and epiregulin (EPR; Figure 1A; Knudsen et al., 2014; Singh et al., 2016). In young adult mice, bulk transcriptomic analysis could resolve their detection across multiple cell types where most of these are expressed by the vasculature, choroid plexus (TGFα), or astrocytes (HB-EGF; Azim et al., 2018). It remains to be determined which of these are altered during aging and are aspects which will be addressed in follow-up studies using the same procedures done in older mice.

The major downstream effectors of EGFR signaling are described in Figure 1A illustrating the RAS/RAF/ERK1-2/STAT3-5 and the PI3K/AKT protein complexes are fundamental regulators of many OL biological processes aside from the EGFR signaling pathway (Ishii et al., 2014, 2019; Azim et al., 2017; Sanz-Rodriguez et al., 2018; Rivera et al., 2021b). The precise interaction of these kinases to the newly identified EGFR-VINCULIN-GELSOLIN-CLDN11 network remains to be resolved.

### EGFR Signaling in Oligodendroglia and Myelination

Recent transcriptomic studies have shed further light on the expression of EGFR and ERRB2–4 in developmental and adult human OL lineage cells demonstrating elevated expression in OPC compared to other CNS cell types (Zhang et al., 2014; Jäkel et al., 2019). *In vivo* gain- and loss-of-function studies underlined the critical importance of EGFR in OLs (Aguirre et al., 2007, 2010). Specifically, overexpression of EGFR enhanced the densities and maturation state of myelinating oligodendrocytes (MOL; Aguirre et al., 2007), which may be owed to sustained AKT phosphorylation in post-mitotic immature OLs (Flores et al., 2000, 2008) *via* the Src homology 2-containing phosphotyrosine phosphatase (SHP2) which integrates EGFR to AKT signaling (Agazie and Hayman, 2003; Zhu et al., 2010; Nocita et al., 2019). Similarly, the Grb2 associated binder 1 (GAB1) is another modulator of PI3K/AKT signaling capable of directing oligodendrogenesis *via* EGFR (Holgado-Madruga et al., 1996; Hayakawa-Yano et al., 2007). *In vitro* studies indicated that EGF interacts with PDGF-AA and FGF to direct early glial progenitors toward the OL fate, suggesting the combinatorial role for these two pathways towards oligodendrogenesis (Yang et al., 2017). Intriguingly, OPCs cultured with EGF in the absence of PDGF-AA are driven to differentiate into MOLs, suggesting a dual role of EGF in the control of oligodendrogenesis and myelination, depending on the activation of other signaling pathways (Yang et al., 2017). Similarly, intraventricular administration of EGF promotes subventricular progenitors to differentiate into OPCs and MOL *in vivo* (Gonzalez-Perez et al., 2009; Galvez-Contreras et al., 2013). Furthermore, overexpression of human-EGFR (hEGFR) in CNP+ OLs leads to an increase in OL densities and remyelination of the corpus callosum following a lesion (Aguirre et al., 2007). However, persistent overexpression of EGFR in OPCs leads to their dramatic enhancement in densities and white matter hyperplasia, although differentiation appears to be hindered (Hayakawa-Yano et al., 2007; Ivkovic et al., 2008; Gonzalez-Perez et al., 2009). Finally, intranasal administration of exogenous heparin-binding EGF (HB-EGF) in a neonatal hypoxia murine model reduced apoptosis of MOL preserving axonal myelination (Scafidi et al., 2014). These results suggest that the control of oligodendrogenesis and myelination requires temporal and cell-specific interplay of EGFR with other unknown signaling pathways. OL lineage cells also express ERBB2–4 which are required depending on the maturation stage (Flores et al., 2000; Park et al., 2001; Yarden and Sliwkowski, 2001; Kim et al., 2003; Schmucker et al., 2003; Makinodan et al., 2012). For example, ERBB2 regulates OL proliferation and differentiation, while ERBB3 and ERBB4 are necessary for maturation and myelination (Park et al., 2001; Schmucker et al., 2003; Roy et al., 2007; Joubert et al., 2010; Makinodan et al., 2012). However, overexpression or excessive activation of ERBBs in defined stages of OLs can lead to inflammation, demyelination, and cell death (Hu et al., 2021). Taken together, EGFR/ERBB signaling has key roles in the regulation of defined stages of OLs and white matter formation in the CNS.

## EGFR as A Potential Therapeutic Strategy in Age-Related White Matter Loss

**Figure 2:**
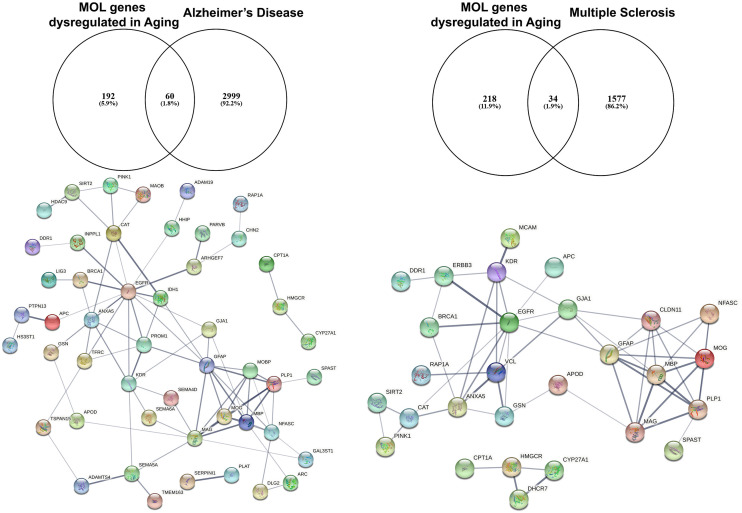
Identification of altered EGFR signaling in aged myelinating oligodendrocytes are further associated as altered genes in Alzheimer’s disease and multiple sclerosis. The aging myelinating OLtranscriptome was interrogated to identify novel associations within disease-specific databases for Alzheimer’s disease and multiple sclerosis. Functional protein–protein prediction analysis identified EGFR at the core of the networks for Alzheimer’s disease and multiple sclerosis (PPI enrichment *p* < 0.0001).

Studies on preclinical murine animal models and human post-mortem tissue have demonstrated downregulation of EGFR in the aging brain (Hiramatsu et al., 1988; Werner et al., 1988). Moreover, EGFR signaling is disturbed in aging and gain and loss of function experiments *in vivo* posit the idea of the presence of undiscovered factors in the aging CNS that limit its efficacy in positively regulating biological processes required for proliferation and differentiation of NSCs (Cochard et al., 2021). To further investigate this, we have interrogated the transcriptome of the aging murine brain (Rivera et al., 2021b) with genes associated with Alzheimer’s Disease (AD) and Multiple Sclerosis (MS) using the DISGENET database (Figure 2; Pinero et al., 2015). These analyses identified 60 AD-associated genes that are altered in aged MOLs, with an apparent EGFR-ANXA5-GSN-APOD axis interconnected with a myelin gene hub; ANXA5 (annexin 5) is involved in pathogenesis through autophagy mechanisms (Iannaccone et al., 2015; Xi et al., 2020), and APOD (apolipoprotein D) is a secreted glycoprotein involved in lipid transport that is linked to AD, MS and other neuroinflammatory diseases (Reindl et al., 2001; Li et al., 2015). In the same way, analysis of oligodendroglial genes altered in aging and associated with MS revealed 34 highly correlated genes with a conserved EGFR-VCL-GSN-APOD network associated with myelin genes. In MS, APOD levels are decreased in sclerotic plaques and elevated during remyelination (Navarro et al., 2018). Hence, identifying small molecules that target these networks has promising therapeutic potential for rejuvenating OPCs in aging contexts. A novel approach is to use genomics and chemical informatics data, most notably the connectivity map (CMAP) and the Library of Integrated Network-based Cellular Signatures (LINCS), which enables the identification of small molecules that counteract disease-specific transcriptional profiles (Azim et al., 2017; Rivera et al., 2022a). In this way, we recently identified the PI3K-Akt inhibitor LY294002 as a potent driver of OPC rejuvenation and myelin repair *in vivo* (Rivera et al., 2022a, b). Notably, transcriptional profiling and signaling pathway activity assays identified EGFR signaling as a target of LY294002 in OPCs and GO analysis of LY294002-responsive oligodendroglial genes indicated a central role for Rhoa at the core of ERBB3 signaling, which regulates oligodendrogenesis *via* RAF-MAPK and PI3K/Akt (Rivera et al., 2022a). These studies demonstrate that transcript-specific targeting using pharmacogenomics approaches streamlines the identification of drugs to target EGFR signaling, and can be readily adapted to probe genes and transcriptional networks of interest for driving rejuvenation and myelin repair.

## Conclusion

In summary, EGFR signaling and its subsequent signaling cascade depends on the combination of ERBB receptors activated. EGFR signaling is central for the OPC self-renewal and their differentiation into MOLs. In the aged brain, there is a decline in the regenerative capacity of OPCs and this is highly correlated with changes in EGFR signaling pathways. Moreover, we have recently shown that targeting PI3K/AKT signaling, which is a key downstream mechanism of EGFR, promotes OPC regeneration and remyelination in an aging context (Rivera et al., 2022a, b). These data support a key role for EGFR as a potential therapeutic target for rejuvenating OPCs and promoting repair in pathologies with age-related contexts, including MS and AD.

## Author Contributions

AR: conceptualization, formal analysis, investigation, methodology, writing—original draft, writing—review and editing. KA: formal analysis, investigation, writing—review and editing. VM and AP: supervision, writing—review and editing. AB and RD: conceptualization, formal analysis, funding acquisition, project administration, resources, supervision, visualization, writing—original draft, writing—review and editing.

## Funding

This work was supported by grants from the Biotechnology and Biological Sciences Research Council (BBSRC, UK) (BB/M029379/1), Medical Research Council (MRC, UK) (MR/P025811/1), MSCA Seal of Excellence @ UNIPD and NVIDIA Hardware Grant, German Research Council (AZ/115/1-1; AZ/115/1-3), and Swiss National Funds (P300PA_171224).

## Conflict of Interest

AR and AB are shareholders of Gliagenesis LTD. The remaining authors declare that the research was conducted in the absence of any commercial or financial relationships that could be construed as a potential conflict of interest.

## Publisher’s Note

All claims expressed in this article are solely those of the authors and do not necessarily represent those of their affiliated organizations, or those of the publisher, the editors and the reviewers. Any product that may be evaluated in this article, or claim that may be made by its manufacturer, is not guaranteed or endorsed by the publisher.
